# 1-Chloro­methyl-1,4-diazo­niabicyclo[2.2.2]octane tetra­chloridocuprate(II)

**DOI:** 10.1107/S1600536811020782

**Published:** 2011-06-11

**Authors:** Tao Rong

**Affiliations:** a Ordered Matter Science Research Center, Southeast University, Nanjing 210096, People’s Republic of China

## Abstract

In the crystal structure of the title compound, (C_7_H_15_ClN_2_)[CuCl_4_], a weak inter­molecular N—H⋯Cl hydrogen bond is observed between the organic dication and the tetrahedral [CuCl_4_]^2−^ anion. The organic dication is distorted, as indicated by the N—C—C—N torsion angles, which range from 16.76 (4) to 19.54 (3)°.

## Related literature

For related 1,4-diaza­bicyclo­[2.2.2]octane tetra­chlorido­cuprate(II) and tetra­chloridocobaltate(II) structures, and related references therein, see: Sun & Qu (2005 ▶); Qu & Sun (2005 ▶). For phase transitions of ferroelectric materials, see: Zhang *et al.* (2008 ▶); Ye *et al.* (2009 ▶).
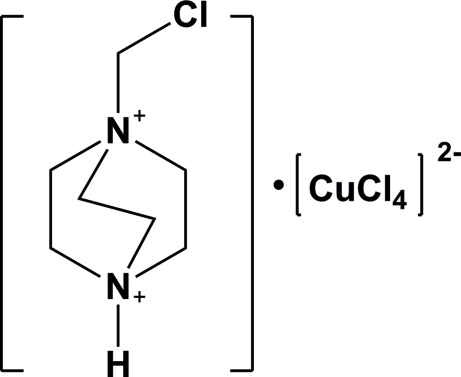


## Experimental

### 

#### Crystal data

(C_7_H_15_ClN_2_)[CuCl_4_]
*M* *_r_* = 368.00
Orthorhombic, 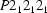

*a* = 9.878 (4) Å
*b* = 11.167 (4) Å
*c* = 12.201 (4) Å
*V* = 1345.9 (8) Å^3^
*Z* = 4
Mo *K*α radiation
μ = 2.59 mm^−1^
*T* = 293 K
0.30 × 0.25 × 0.20 mm

#### Data collection

Rigaku Mercury2 diffractometer
Absorption correction: multi-scan (*CrystalClear*; Rigaku, 2005 ▶) *T*_min_ = 0.465, *T*_max_ = 0.596
6091 measured reflections
3072 independent reflections
2865 reflections with *I* > 2σ(*I*)
*R*_int_ = 0.032

#### Refinement

*R*[*F* ^2^ > 2σ(*F* ^2^)] = 0.022
*wR*(*F* ^2^) = 0.055
*S* = 1.01
3072 reflections
136 parameters
H-atom parameters constrained
Δρ_max_ = 0.39 e Å^−3^
Δρ_min_ = −0.36 e Å^−3^
Absolute structure: Flack (1983 ▶), with 1298 Friedel pairs
Flack parameter: 0.006 (11)

### 

Data collection: *CrystalClear* (Rigaku, 2005 ▶); cell refinement: *CrystalClear*; data reduction: *CrystalClear*; program(s) used to solve structure: *SHELXS97* (Sheldrick, 2008 ▶); program(s) used to refine structure: *SHELXL97* (Sheldrick, 2008 ▶); molecular graphics: *ORTEP-3* (Farrugia, 1997 ▶); software used to prepare material for publication: *SHELXL97*.

## Supplementary Material

Crystal structure: contains datablock(s) I, global. DOI: 10.1107/S1600536811020782/si2354sup1.cif

Structure factors: contains datablock(s) I. DOI: 10.1107/S1600536811020782/si2354Isup2.hkl

Additional supplementary materials:  crystallographic information; 3D view; checkCIF report

## Figures and Tables

**Table 1:** Selected bond lengths (Å)

Cl2—Cu1	2.2537 (8)
Cl3—Cu1	2.2539 (9)
Cl4—Cu1	2.2559 (9)
Cl5—Cu1	2.2088 (11)

**Table 2:** Hydrogen-bond geometry (Å, °)

*D*—H⋯*A*	*D*—H	H⋯*A*	*D*⋯*A*	*D*—H⋯*A*
N2—H2*C*⋯Cl2	0.91	2.60	3.270 (2)	131
N2—H2*C*⋯Cl3	0.91	2.54	3.252 (2)	136

**Fig. 1.**
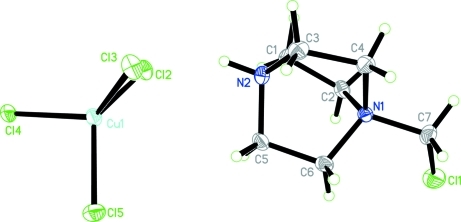
The molecular structure of the title compound. Displacement ellipsoids are drawn at the 30% probability level.

**Fig. 2.**
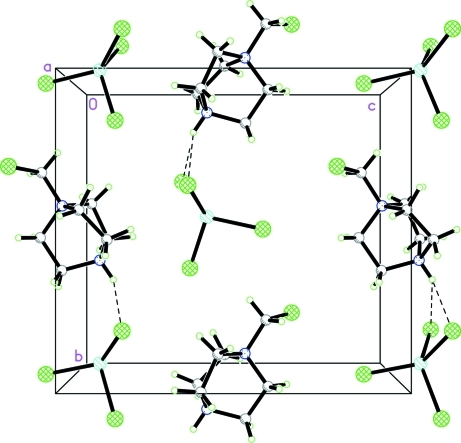
A view of a packing section of the title compound, stacking along the c axis. Dashed lines indicate hydrogen bonds.

## supplementary crystallographic information

### Comment

The title compound (I), (Fig. 1), consists of protonated 1-(chloridomethyl)-1,4-diazabicyclo[2.2.2]octane-1,4-diium dications and [CuCl_4_]^2-^ anions. The organic dication is distorted, as indicated by the N—C—C—N torsion angles, which range from 16.76 (4) to 19.54 (3)°. In the structure of 1,4-dimethyl-1,4-diazonia[2.2.2]octane tetrachloridocuprate(II), of two independent dications one is almost undistorted with torsion angles between 0.6 (6) and 0.9 (5)°, whereas the other dication is distorted exhibiting torsion angles in the range of 5.5 (5) and 7.9 (5)° (Sun & Qu, 2005). In the isotypic cobalt(II) structure (Qu & Sun, 2005), two independent dications are slightly distorted with torsion angles range between 3.0 (4) and 8.7 (4)°. The [CuCl_4_]^2-^ anion in (I) possesses typical Cu—Cl bonds and its lengths range from 2.209 (1) to 2.2559 (9) Å (Table 1), while the Cl—Cu—Cl angles range from 95.98 (4) to 132.85 (3)°. The bifurcated N—H···(Cl,Cl) hydrogen bonds (Table 2) between the organic dications and the [CuCl_4_]^2-^ anions contribute to the stability of crystal packing (Fig. 2).

The study of ferroelectric materials has received much attention. Some materials have predominantly dielectric-ferroelectric performance.The title compound was studied as part of our work to obtain potential ferroelectric phase transition materials. Unluckily, the compound has no dielectric anomalies in the temperature range 93–453 K, suggesting that it might be only a paraelectric (Zhang *et al.*, 2008; Ye *et al.*, 2009).

### Experimental

1, 4-diazabicyclo [2.2.2]octane (5.6 g, 0.05 mol) was added in dichloromethane (20 ml) and the mixture was refluxed for 8 h. On standing for about 16 h at room temperature, the white precipitate of 1-(chloridomethyl)-1,4-diazabicyclo[2.2.2]octan-1-ium chloride was obtained.

The title compound was synthesized by adding a solution of 1-(chloridomethyl)-1,4-diazabicyclo[2.2.2]octan-1-ium chloride (1.97 g, 10 mmol) in HCl (37%, 20 ml) to a solution of CuCl_2_ (8 mmol) in 20 ml H_2_O. After a few weeks, brown hygroscopic block crystals of the title compound were obtained on slow evaporation of the solvent.

### Refinement

Positional parameters of all H atoms bonded to C and N atoms were calculated geometrically and were allowed to ride on the C and N atoms to which they are bonded, with respective C—H and N—H distances of 0.97 Å and 0.91 Å and with *U*_iso_(H) = 1.2*U*_eq_(C, N).

### Crystal data

**Table:**

(C_7_H_15_ClN_2_)[CuCl_4_]	*F*(000) = 740
*M**_r_* = 368.00	*D*_x_ = 1.816 Mg m^−^^3^
Orthorhombic, *P*2_1_2_1_2_1_	Mo *K*α radiation, λ = 0.71073 Å
Hall symbol: P 2ac 2ab	Cell parameters from 4288 reflections
*a* = 9.878 (4) Å	θ = 2.5–27.5°
*b* = 11.167 (4) Å	µ = 2.59 mm^−^^1^
*c* = 12.201 (4) Å	*T* = 293 K
*V* = 1345.9 (8) Å^3^	Block, brown
*Z* = 4	0.30 × 0.25 × 0.20 mm

### Data collection

**Table:**

Rigaku Mercury2 diffractometer	3072 independent reflections
Radiation source: fine-focus sealed tube	2865 reflections with *I* > 2σ(*I*)
graphite	*R*_int_ = 0.032
Detector resolution: 28.5714 pixels mm^-1^	θ_max_ = 27.5°, θ_min_ = 2.5°
CCD profile fitting scans	*h* = −12→12
Absorption correction: multi-scan (*CrystalClear*; Rigaku, 2005)	*k* = −14→14
*T*_min_ = 0.465, *T*_max_ = 0.596	*l* = −15→15
6091 measured reflections	

### Refinement

**Table:**

Refinement on *F*^2^	Secondary atom site location: difference Fourier map
Least-squares matrix: full	Hydrogen site location: inferred from neighbouring sites
*R*[*F*^2^ > 2σ(*F*^2^)] = 0.022	H-atom parameters constrained
*wR*(*F*^2^) = 0.055	*w* = 1/[σ^2^(*F*_o_^2^) + (0.0267*P*)^2^] where *P* = (*F*_o_^2^ + 2*F*_c_^2^)/3
*S* = 1.01	(Δ/σ)_max_ = 0.001
3072 reflections	Δρ_max_ = 0.39 e Å^−^^3^
136 parameters	Δρ_min_ = −0.36 e Å^−^^3^
0 restraints	Absolute structure: Flack (1983), with 1298 Friedel pairs
Primary atom site location: structure-invariant direct methods	Flack parameter: 0.006 (11)

### Special details

**Table:**

Refinement. Refinement of *F*^2^ against ALL reflections. The weighted *R*-factor *wR* and goodness of fit *S* are based on *F*^2^, conventional *R*-factors *R* are based on *F*, with *F* set to zero for negative *F*^2^. The threshold expression of *F*^2^ > σ(*F*^2^) is used only for calculating *R*-factors(gt) *etc*. and is not relevant to the choice of reflections for refinement. *R*-factors based on *F*^2^ are statistically about twice as large as those based on *F*, and *R*-factors based on ALL data will be even larger.

### Fractional atomic coordinates and isotropic or equivalent isotropic displacement parameters (Å^2^)

**Table:**

	*x*	*y*	*z*	*U*_iso_*/*U*_eq_	
C1	0.4179 (3)	1.0325 (2)	0.3874 (2)	0.0310 (6)	
H1A	0.4300	0.9982	0.3150	0.037*	
H1B	0.3385	1.0836	0.3859	0.037*	
C2	0.3989 (3)	0.9330 (2)	0.4717 (2)	0.0267 (6)	
H2A	0.3256	0.9533	0.5212	0.032*	
H2B	0.3760	0.8586	0.4350	0.032*	
C3	0.6657 (3)	1.0334 (2)	0.4017 (2)	0.0303 (6)	
H3A	0.7419	1.0735	0.4359	0.036*	
H3B	0.6844	1.0253	0.3240	0.036*	
C4	0.6448 (3)	0.9099 (2)	0.4533 (2)	0.0286 (6)	
H4A	0.6235	0.8517	0.3968	0.034*	
H4B	0.7269	0.8845	0.4902	0.034*	
C5	0.5303 (3)	1.1391 (2)	0.5373 (2)	0.0288 (6)	
H5A	0.4426	1.1748	0.5519	0.035*	
H5B	0.5998	1.1973	0.5551	0.035*	
C6	0.5486 (3)	1.0273 (2)	0.6064 (2)	0.0282 (6)	
H6A	0.6388	1.0263	0.6380	0.034*	
H6B	0.4834	1.0269	0.6659	0.034*	
C7	0.5329 (3)	0.8043 (2)	0.6006 (2)	0.0336 (6)	
H7A	0.6137	0.8040	0.6457	0.040*	
H7B	0.5382	0.7367	0.5510	0.040*	
Cl1	0.39073 (9)	0.78795 (7)	0.68465 (6)	0.0493 (2)	
N1	0.5285 (2)	0.91796 (17)	0.53523 (16)	0.0212 (4)	
N2	0.5402 (2)	1.10429 (17)	0.41845 (17)	0.0255 (4)	
H2C	0.5438	1.1716	0.3765	0.031*	
Cl2	0.36056 (6)	1.33367 (5)	0.33718 (6)	0.03068 (15)	
Cl3	0.69739 (6)	1.35292 (6)	0.36324 (6)	0.03568 (16)	
Cl4	0.42453 (7)	1.63249 (5)	0.36275 (5)	0.03390 (16)	
Cl5	0.55993 (8)	1.49699 (6)	0.59012 (6)	0.04221 (18)	
Cu1	0.51283 (3)	1.45593 (3)	0.41718 (3)	0.02648 (9)	

### Atomic displacement parameters (Å^2^)

**Table:**

	*U*^11^	*U*^22^	*U*^33^	*U*^12^	*U*^13^	*U*^23^
C1	0.0292 (13)	0.0314 (14)	0.0325 (15)	−0.0047 (12)	−0.0084 (11)	0.0029 (11)
C2	0.0233 (13)	0.0228 (13)	0.0339 (14)	−0.0040 (10)	−0.0025 (11)	−0.0032 (11)
C3	0.0237 (12)	0.0379 (14)	0.0294 (14)	0.0008 (12)	0.0058 (11)	0.0033 (12)
C4	0.0255 (13)	0.0314 (13)	0.0289 (14)	0.0070 (12)	0.0048 (11)	0.0014 (11)
C5	0.0342 (15)	0.0215 (12)	0.0305 (13)	−0.0036 (12)	0.0031 (12)	−0.0053 (10)
C6	0.0338 (14)	0.0260 (13)	0.0248 (14)	−0.0091 (11)	0.0016 (10)	−0.0047 (10)
C7	0.0426 (16)	0.0270 (13)	0.0312 (15)	−0.0047 (12)	−0.0017 (13)	0.0070 (11)
Cl1	0.0659 (6)	0.0429 (4)	0.0392 (4)	−0.0177 (4)	0.0166 (4)	0.0024 (3)
N1	0.0226 (10)	0.0194 (9)	0.0217 (10)	−0.0025 (8)	−0.0004 (9)	−0.0012 (8)
N2	0.0260 (10)	0.0214 (10)	0.0292 (11)	−0.0023 (9)	−0.0003 (10)	0.0027 (9)
Cl2	0.0259 (3)	0.0249 (3)	0.0412 (4)	−0.0006 (3)	−0.0059 (3)	−0.0022 (3)
Cl3	0.0241 (3)	0.0314 (3)	0.0516 (4)	0.0000 (3)	−0.0010 (3)	−0.0042 (3)
Cl4	0.0505 (4)	0.0211 (3)	0.0302 (3)	0.0052 (3)	−0.0030 (3)	−0.0007 (3)
Cl5	0.0568 (5)	0.0438 (4)	0.0260 (3)	0.0048 (4)	−0.0066 (3)	−0.0006 (3)
Cu1	0.02908 (17)	0.02223 (14)	0.02814 (16)	0.00119 (14)	−0.00354 (14)	−0.00133 (13)

### Geometric parameters (Å, °)

**Table:**

C1—N2	1.499 (3)	C5—C6	1.518 (3)
C1—C2	1.526 (3)	C5—H5A	0.9700
C1—H1A	0.9700	C5—H5B	0.9700
C1—H1B	0.9700	C6—N1	1.511 (3)
C2—N1	1.506 (3)	C6—H6A	0.9700
C2—H2A	0.9700	C6—H6B	0.9700
C2—H2B	0.9700	C7—N1	1.499 (3)
C3—N2	1.484 (3)	C7—Cl1	1.748 (3)
C3—C4	1.530 (3)	C7—H7A	0.9700
C3—H3A	0.9700	C7—H7B	0.9700
C3—H3B	0.9700	N2—H2C	0.9100
C4—N1	1.526 (3)	Cl2—Cu1	2.2537 (8)
C4—H4A	0.9700	Cl3—Cu1	2.2539 (9)
C4—H4B	0.9700	Cl4—Cu1	2.2559 (9)
C5—N2	1.504 (3)	Cl5—Cu1	2.2088 (11)
			
N2—C1—C2	108.56 (19)	N1—C6—C5	109.22 (19)
N2—C1—H1A	110.0	N1—C6—H6A	109.8
C2—C1—H1A	110.0	C5—C6—H6A	109.8
N2—C1—H1B	110.0	N1—C6—H6B	109.8
C2—C1—H1B	110.0	C5—C6—H6B	109.8
H1A—C1—H1B	108.4	H6A—C6—H6B	108.3
N1—C2—C1	108.86 (19)	N1—C7—Cl1	112.18 (19)
N1—C2—H2A	109.9	N1—C7—H7A	109.2
C1—C2—H2A	109.9	Cl1—C7—H7A	109.2
N1—C2—H2B	109.9	N1—C7—H7B	109.2
C1—C2—H2B	109.9	Cl1—C7—H7B	109.2
H2A—C2—H2B	108.3	H7A—C7—H7B	107.9
N2—C3—C4	108.13 (19)	C7—N1—C2	113.10 (19)
N2—C3—H3A	110.1	C7—N1—C6	111.95 (19)
C4—C3—H3A	110.1	C2—N1—C6	108.52 (19)
N2—C3—H3B	110.1	C7—N1—C4	106.10 (19)
C4—C3—H3B	110.1	C2—N1—C4	108.03 (19)
H3A—C3—H3B	108.4	C6—N1—C4	108.98 (18)
N1—C4—C3	108.55 (19)	C3—N2—C1	110.7 (2)
N1—C4—H4A	110.0	C3—N2—C5	109.0 (2)
C3—C4—H4A	110.0	C1—N2—C5	109.21 (19)
N1—C4—H4B	110.0	C3—N2—H2C	109.3
C3—C4—H4B	110.0	C1—N2—H2C	109.3
H4A—C4—H4B	108.4	C5—N2—H2C	109.3
N2—C5—C6	108.37 (19)	Cl5—Cu1—Cl2	132.85 (3)
N2—C5—H5A	110.0	Cl5—Cu1—Cl3	102.39 (3)
C6—C5—H5A	110.0	Cl2—Cu1—Cl3	95.98 (4)
N2—C5—H5B	110.0	Cl5—Cu1—Cl4	100.44 (3)
C6—C5—H5B	110.0	Cl2—Cu1—Cl4	98.27 (4)
H5A—C5—H5B	108.4	Cl3—Cu1—Cl4	132.29 (3)
			
N2—C1—C2—N1	−16.8 (3)	C5—C6—N1—C4	68.1 (2)
N2—C3—C4—N1	−19.5 (3)	C3—C4—N1—C7	−167.6 (2)
N2—C5—C6—N1	−16.8 (3)	C3—C4—N1—C2	70.9 (2)
Cl1—C7—N1—C2	−52.6 (2)	C3—C4—N1—C6	−46.9 (3)
Cl1—C7—N1—C6	70.4 (2)	C4—C3—N2—C1	−47.8 (3)
Cl1—C7—N1—C4	−170.83 (17)	C4—C3—N2—C5	72.3 (2)
C1—C2—N1—C7	−166.0 (2)	C2—C1—N2—C3	69.9 (3)
C1—C2—N1—C6	69.1 (2)	C2—C1—N2—C5	−50.1 (3)
C1—C2—N1—C4	−48.9 (2)	C6—C5—N2—C3	−51.1 (3)
C5—C6—N1—C7	−174.9 (2)	C6—C5—N2—C1	70.0 (3)
C5—C6—N1—C2	−49.3 (2)		

### Hydrogen-bond geometry (Å, °)

**Table:**

*D*—H···*A*	*D*—H	H···*A*	*D*···*A*	*D*—H···*A*
N2—H2C···Cl2	0.91	2.60	3.270 (2)	131.
N2—H2C···Cl3	0.91	2.54	3.252 (2)	136.
